# Parasitism in early life: environmental conditions shape within-brood variation in responses to infection

**DOI:** 10.1002/ece3.1192

**Published:** 2014-08-18

**Authors:** Hanna M V Granroth-Wilding, Sarah J Burthe, Sue Lewis, Thomas E Reed, Katherine A Herborn, Mark A Newell, Emi A Takahashi, Francis Daunt, Emma J A Cunningham

**Affiliations:** 1 Centre for Immunity, Infection and Evolution, Institute of Evolutionary Biology, School of Biology, University of Edinburgh, Ashworth LaboratoriesKing's Buildings, West Mains Rd, Edinburgh, EH9 3JT, U.K; 2 NERC Centre for Ecology & Hydrology, Bush EstatePenicuik, Midlothian, EH26 0QB, U.K; 3 Institute of Evolutionary Biology, School of Biology, University of Edinburgh, Ashworth LaboratoriesKing's Buildings, West Mains Rd, Edinburgh, EH9 3JT, U.K; 4 School of Biological, Earth & Environmental Sciences, University College CorkCork, Ireland; 5 Institute of Biodiversity, Animal Health & Comparative Medicine, University of GlasgowBearsden Road, Glasgow, G61 1QH, U.K

**Keywords:** Brood conflict, climate change, environmental variability, host, individual differences, ivermectin, nematode, parasite, seabird, sibling competition

## Abstract

Parasites play key ecological and evolutionary roles through the costs they impose on their host. In wild populations, the effect of parasitism is likely to vary considerably with environmental conditions, which may affect the availability of resources to hosts for defense. However, the interaction between parasitism and prevailing conditions is rarely quantified. In addition to environmental variation acting on hosts, individuals are likely to vary in their response to parasitism, and the combined effect of both may increase heterogeneity in host responses. Offspring hierarchies, established by parents in response to uncertain rearing conditions, may be an important source of variation between individuals. Here, we use experimental antiparasite treatment across 5 years of variable conditions to test how annual population productivity (a proxy for environmental conditions) and parasitism interact to affect growth and survival of different brood members in juvenile European shags (*Phalacrocorax aristotelis*). In control broods, last-hatched chicks had more plastic growth rates, growing faster in more productive years. Older siblings grew at a similar rate in all years. Treatment removed the effect of environment on last-hatched chicks, such that all siblings in treated broods grew at a similar rate across environmental conditions. There were no differences in nematode burden between years or siblings, suggesting that variation in responses arose from intrinsic differences between chicks. Whole-brood growth rate was not affected by treatment, indicating that within-brood differences were driven by a change in resource allocation between siblings rather than a change in overall parental provisioning. We show that gastrointestinal parasites can be a key component of offspring's developmental environment. Our results also demonstrate the value of considering prevailing conditions for our understanding of parasite effects on host life-history traits. Establishing how environmental conditions shape responses to parasitism is important as environmental variability is predicted to increase.

## Introduction

Parasites play a key role in many ecological and evolutionary processes through the costs they impose on their host (Sheldon & Verhulst 1996; Clayton and Moore 1997; Norris and Evans 2000; Hudson et al. 2002; Sandland and Minchella 2003). However, in wild populations, the effect of parasitism may vary considerably with external conditions, potentially having a greater effect when conditions are poor because hosts have fewer resources to deal with infection. Many environmental factors are predicted to become more variable in the near future due to climatic change (Coumou and Rahmstorf 2012). Understanding how this extrinsic variability interacts with parasitism to influence an organism's life history is therefore critical to understanding the full impact of both factors on populations. However, in studies of wild hosts, variation in how hosts cope with infection is rarely considered explicitly in the context of prevailing environmental conditions (Sandland & Minchella 2003; Wolinska & King 2009; Boughton et al. 2011). Instead, parasite manipulation studies commonly interpret environmental variability as noise to which conclusions should be robust, rather than an informative aspect of host responses to infection. Qualitative interannual differences in the effects of parasite manipulation in wild hosts have been observed (e.g., Heeb et al. 1999; Stien et al. 2002; Knowles et al. 2010b), and the importance of environment to host–parasite interactions is well acknowledged in laboratory systems (Luong and Polak 2007; Wolinska & King 2009; Vale et al. 2011), yet we know of no study that incorporates quantitative measures of interannual environmental variation to examine the consequences of parasitism in a wild, free-ranging population.

Individuals in a population are likely to vary in their response to environmental conditions, their susceptibility to parasite infection, and their subsequent ability to deal with an established infection (Schmid-Hempel & Koella 1994; Shaw, Grenfell & Dobson 1998; Sandland and Minchella 2003; Lewis et al. 2009). This variation among individuals is likely to be particularly pronounced in juveniles, because parental investment patterns may vary strategically with environmental conditions to maximize lifetime reproductive success (Temme & Charnov 1987; Forbes 2009). For example, parents with multiple offspring may bias provisioning to ensure that core young obtain sufficient resources to survive when conditions are poor (Lack 1947; Mock and Forbes 1995; Forbes et al. 2002; Hudson and Trillmich 2007; Forbes 2009). Much of the research in this area has been carried out in birds, where this bias often stems from within-brood asymmetry in size, commonly set up by asynchronous hatching of eggs and differences in the hormonal environment of offspring (Stenning 1996; Bonabeau et al. 1998; Groothuis et al. 2005). Levels of maternal antibodies and nutrients may also differ in relation to laying order and offspring sex (Royle et al. 1999; Pihlaja et al. 2006; Hasselquist and Nilsson 2009; Martyka et al. 2011). Inherent differences between offspring therefore exist that could influence both their susceptibility and tolerance to parasitism, leading to complex, nonadditive effects of parasitism under different environmental conditions (Forbes 1993; Bize et al. 2006; Knowles et al. 2010a).

Furthermore, the developmental environment may also influence the value of the whole brood to parents. There may therefore be variation in the total amount of food that parents provide to the nest as well as allocation between brood members (Godfray and Johnstone 2000; Parker et al. 2002). While offspring parasitism has been shown to alter overall parental provisioning (Christe et al. 1996; Tripet & Richner 1997; Hurtrez-Boussès et al. 1998), little is yet known about whether it can influence resource allocation among a brood.

A number of studies that manipulate ectoparasite loads have demonstrated an impact of infection on a range of traits across family members, showing that parasitism in the nest can be detrimental to the development of individual offspring (O'Brien and Dawson 2009), to the success of the whole brood (Christe et al. 1996), and to parents' future breeding success (Bize et al. 2004; Fitze et al. 2004). These effects could be driven by various behavioral mechanisms of intrafamilial conflict. For example, ectoparasitism has been shown to influence both chick signaling and parental provisioning in great tits *Parus major*, where removal of biting hen fleas in the nest decreased both chick begging rate and the father's provisioning rate (Christe et al. 1996). However, many ectoparasites are mobile and redistribute themselves between chicks and parents to feed and disperse (Tripet & Richner 1999). It is therefore difficult to completely isolate the effect of a particular individual's parasite load, as removing parasites from one family member may alter the parasite load of others (Bize et al. 2004; Fitze et al. 2004; Gallizzi et al. 2008; Roulin et al. 2003). In contrast, endoparasites are discretely distributed between hosts, allowing the direct costs to the host and the indirect effects on other family members to be distinguished. Separating these effects would be a major step in advancing our understanding of how individual differences in responses to parasitism are affected by environmental conditions. This will be key when considering how effects of parasitism may scale to affect different populations of varying composition as they face environmental change.

**Figure 1:**
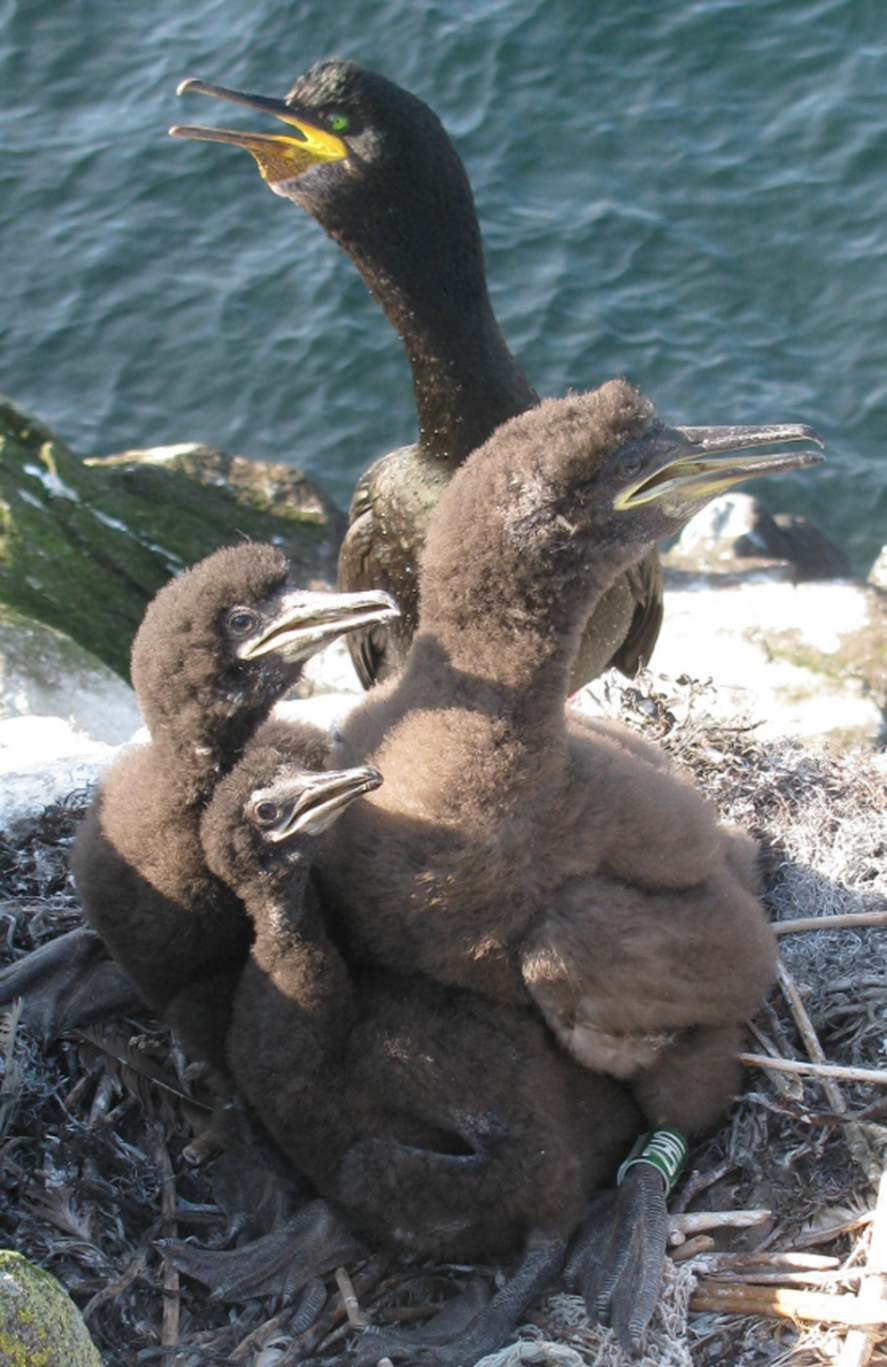
A brood of asynchronously hatched European shags (*Phalacrocorax aristotelis*), aged c. 25 days, with an attending parent.

In this study, we examine the effect of annual population productivity (a proxy of prevailing environmental conditions) on the consequences of parasitism in juvenile European shags, *Phalacrocorax aristotelis* (Fig.1). Individuals of this species are infected with gastrointestinal nematodes from the fish they eat (Anderson 2000; Hoberg 2005; Fagerholm and Overstreet 2008), and there is a high prevalence of infection among adults and juveniles (Reed et al. 2012; Burthe et al. 2013; Granroth-Wilding 2013). Shag chicks hatch asynchronously, and chick survival varies considerably among years. Last-hatched chicks show lower survival on average (Amundsen and Stokland 1988) and therefore potentially more variable responses to environmental conditions. Here, we experimentally manipulate parasite loads over 5 years of variable conditions to investigate the effect of gastrointestinal nematode infection on individual chick growth rate and survival in a system where we can disentangle the confounding effects of parasite distributions between related individuals. We also investigate whether these differences arise as a result of changes in parental resource provisioning to the whole brood or to changes in how resources are allocated to different members of a brood.

## Methods

### Study site and species

This experiment was carried out on the breeding population of shags on the Isle of May National Nature Reserve, southeast Scotland (56°11 N, 2°33 W), during the breeding seasons (April–July) in 2006, 2007, 2010, 2011, and 2012. Shags lay a modal clutch of three eggs (range 1-4), each 3 days apart (Snow 1960; Granroth-Wilding 2013). In three egg clutches (78% of clutches in this population), the first egg is the smallest, the second egg is the largest, and the third is generally intermediate in size (Coulson et al. 1969). Incubation begins when the second egg is laid, such that the first two eggs hatch within 24 h of one another (the A & B chicks), while the third hatches c. 2 days later (the C chick) (Potts et al. 1980; Stokland & Amundsen 1988, pers. obs.). This hatching asynchrony creates a size hierarchy where the C chick remains smaller than its older siblings halfway through chick rearing despite the fact that it typically comes from a larger egg than its A egg counterpart (across the 5 years, at age 25 days of a 50-day nestling period, C chick 9% smaller, *P* < 0.001). Chick mortality is highest in the first 10 days after hatching (Daunt et al. 1999) and generally higher for last-hatched chicks (Amundsen and Stokland 1988; this study). Males grow faster than females in this sexually dimorphic species (Daunt et al. 2001a).

Sampling of both adults and chicks in this population has demonstrated a very high prevalence of infection of parasitic gastrointestinal ascaridoid nematodes, predominantly *Contracaecum rudolphii* (68 of 68 adults endoscoped and 31 of 31 chicks over 10 days old dissected following natural mortality), although, burdens vary substantially between individuals (Reed et al. 2012; Burthe et al. 2013; Granroth-Wilding 2013). Seabirds are the definitive hosts for *C. rudolphii* (Anderson 2000; Fagerholm and Overstreet 2008). Adult worms at this stage release eggs into the marine environment via the bird's feces, which hatch and pass through paratenic crustacean and fish hosts to enter the bird's proventriculus where they feed on food ingested by the host (Anderson 2000; Abollo et al. 2001; Fagerholm and Overstreet 2008). Chicks are infected by larval worms in the regurgitated fish they are fed by their parents; direct transmission of adult worms dislodged from the parents' proventriculus may also occur (Hoberg 2005; Fagerholm and Overstreet 2008; Granroth-Wilding 2013). Characteristically of macroparasites, nematode infections in seabirds are rarely lethal (Clayton and Moore 1997; Hoberg 2005; Fagerholm and Overstreet 2008), but they impose costs through direct competition with hosts for their fish prey and damage such as inflammation, tissue necrosis, and secondary bacterial infections at attachment sites (Abollo et al. 2001; Hoberg 2005; S. Burthe & H. Granroth-Wilding, pers. obs.). Shags also host biting lice *Eidemanniella pellucida*, but previous work has found no evidence for an effect of lice on chick growth or survival (Daunt et al. 2001b).

Our study years differed markedly in annual population productivity (Table1), measured as the average number of fledged young per incubated nest in a series of unmanipulated, long-term monitoring plots on the Isle of May, henceforth “productivity.” In the last decade (2002–2012), productivity has ranged from 0.25 to 2.04 fledged chicks per nest (Newell et al. 2012). In shags, as in other seabirds, productivity is best explained by models that integrate multiple environmental factors, including food availability (quality and abundance of sandeel *Ammodytes marinus*, the shag's principal prey: Frederiksen et al. 2006; Burthe et al. 2012), climate (sea surface temperature: Burthe et al. 2012), and weather (rain and wind: Aebischer 1993). These combined measures have been shown to capture the environmental variability that is relevant to a shag's reproductive decisions better than any single measure on its own (Frederiksen et al. 2006; Burthe et al. 2012). We therefore used productivity as an annual proxy for environmental conditions (sensu Danchin et al. 1998; Wilson et al. 2006; Reed et al. 2008b; Bogdanova et al. 2014).

**Table 1:** Sample sizes of control nests, drug-treated nests, and chicks with growth rate data, mean growth rate, and mean productivity in each year of the study. All nests contained three chicks at treatment. Productivity is the mean number of chicks fledged per incubated nest at undisturbed monitoring plots located around the study site.

Year	Control nests	Drug-treated nests	Chicks with growth rate data	Mean growth rate (g/day)	Productivity (chicks/nest)
2006	18	20	109	54.4	1.22 ± 0.11
2007	12	9	46	51.2	1.07 ± 0.12
2010	13	23	107	54.5	2.04 ± 0.14
2011	8	8	47	57.2	1.52 ± 0.11
2012	11	9	48	53.8	1.18 ± 0.10
Total	62	69	357	Mean: 54.2	Mean: 1.41

### Antiparasite treatment experiment

#### Ethics statement

All treatment doses were within an empirically established safe range for adult shags (Reed et al. 2008a; Burthe et al. 2013) and have been previously used on chicks with no negative consequences on survival or growth rate (Reed et al. 2012). All blood sampling and drug administration was carried out under UK Home Office license with full ethical approval.

#### Experimental Procedure

In each experimental breeding season, we compared the effect of treatment on individually marked chicks in antiparasite-treated broods to control broods of three chicks. All study nests were monitored daily from the colony-wide onset of laying to obtain laying dates, which were used to predict hatching date, assuming a mean incubation period of 36 days (Potts et al. 1980). Toward the end of incubation, nests were visited every 1–2 days to obtain an accurate hatching date and hatching order for each chick. Hatchlings were blood sampled for molecular sexing (Griffiths et al. 1996) and individually marked using colored wool or electrical tape, which was replaced regularly until permanent metal rings could be fitted at age c.15 days.

Treatment was carried out when the oldest chick in a brood was 10–14 days old, when all brood members are at an early stage in the linear growth phase. Treatments were assigned randomly, matching control and drug-treated nests for hatch date and colony area. Only broods with three chicks still alive at the point of treatment were used in the experiment. In broods that were assigned to the treatment group, all chicks were simultaneously treated by subcutaneous injection with 0.05 mL of 1% wt/vol ivermectin (Panomec© by Merial UK), a broad-spectrum antihelminthic. Control broods were left untreated (2006) or sham-treated with 0.05 mL distilled water (2007) or saline (2010–2012). Previous studies have found no difference between sham-treated and unmanipulated controls in any of the dependent variables investigated (Reed et al. 2012).

At treatment, chicks were assigned ranks A, B, or C in order of decreasing size. Size at this age correctly identifies the last-hatched chick in 90% of cases (120 of a subset of 134 nests with accurate hatching order for all chicks), and mass asymmetry is likely to be a key driver of within-brood dynamics (Reed et al. 2012). Differences in recommended drug volume as a proportion of body mass between chicks of different weights were so small they did not allow for accurate dose adjustment. Previous parasite treatment studies in our system have shown that within-brood differences in response to fixed volume treatment were not influenced by mass differences at treatment (Reed et al. 2012). However, to ensure that any dosing differences did not bias our observed effects, we controlled statistically for differences between chicks in dose as a proportion of mass.

All chicks in each nest were weighed every 4–7 days until the oldest chick was aged approximately 28-30 days, the end of the linear growth phase. Weights were measured to the nearest 0.5 g for chicks up to 50 g, 1 g up to 300 g, 2.5 g up to 600 g, 5 g up to 1000 g, and 10 g up to 2000 g. After the linear growth period, nests were monitored regularly for chick survival until fledging at ∼50 days (Snow 1960).

#### Worm burdens

To assess whether siblings had different worm burdens either pre- or post-treatment, we collected fecal samples every time a chick excreted during handling and counted the number of nematode eggs in it as a proxy for parasite burden (fecal egg counts or FECs; Seivwright et al. 2004; Atkinson et al. 2009). We had sufficient fecal material for quantitative analysis before and after treatment in 2010, where we obtained samples from 60 chicks in 34 nests before treatment (29 controls, 31 drug-treated) and from 102 chicks in 42 nests after treatment (47 controls and 55 drug-treated), of which 54 chicks in 33 nests had both before- and after-treatment samples (28 controls, 26 drug-treated). Samples were classified as pre- or post-treatment, with a spread of ages (15–25 days) in the post-treatment group. We did not have sufficient fecal material to conduct this comparison in all years, but we were able to examine interannual differences in worm burdens in control groups in 2010, 2011, and 2012. For this 3-year comparison, we obtained post-treatment samples from 119 chicks in 65 control nests.

Samples were frozen and stored at −20°C or in a solution of dimethyl sulfoxide, EDTA, and sodium chloride (DESS) (Yoder et al. 2006; Seutin, White & Boag 1991) at room temperature. Storage had no detectable effect on egg count (negative binomial model, *χ* = 1.57, df = 1, *P* = 0.211; see statistical methods below). FECs were obtained using a flotation technique (adjusted from Bowman and Georgi 2009). The sample was mixed well with concentrated salt/sugar solution at a ratio of 20 mL solution for 1 g of feces and left for at least 60 sec to allow most of the organic debris to settle. Using a pipette, the sample was then mixed gently without disturbing the layer of debris and an aliquot of 0.15 mL taken while raising the pipette up through the liquid. This sample was placed in a McMaster slide, and all nematode eggs under the grid were counted under a light microscope at 40× magnification. This was repeated for three aliquots from each sample, totaling 0.023 g of feces.

#### Statistical analysis

We assessed the effect of antiparasite treatment on the growth rate (g/day) and survival of individual chicks and the combined growth rate of all siblings following treatment. These responses reflect different aspects of how parasitism might affect broods: We expect differences in growth rate among nest-mates to reflect how resources are allocated among siblings, and whole-brood growth rate to reflect total parental provisioning. All analyses were conducted in R version 2.13.1 (R Development Core Team 2011). All models investigating effects on individual chicks included nest as a random factor to account for the nonindependence of chicks in a brood. Apart from the FEC analysis, all models were linear or generalized linear mixed models, fitted using the packages nlme (Pinheiro et al. 2012) and lme4 (Bates et al. 2011), respectively, except whole-brood growth rate, which was modeled using simple linear models in the package stats (R Development Core Team 2011). All parameter estimates and effect sizes are presented as mean ± 1 standard error. All model selection used Akaike's information criterion (AIC) to determine the model that best fit the data (Burnham and Anderson 2002).

#### Fecal egg counts

We modeled FECs using a negative binomial distribution in the package MASS (Venables & Ripley 2002) to account for their heavily skewed distribution, with many zeros and few very high counts, common in parasitological data (Shaw et al. 1998). Pre- and post-treatment samples from 2010 were analyzed separately to account for partial resampling of chicks. For each, we tested the effect of rank, treatment, and a rank-by-treatment interaction on burdens. To examine interannual variability in worm burdens, we tested the effect of year as a factor (too few years to robustly fit productivity as a covariate) on fecal egg counts in post-treatment control chicks across the 3 years (2010, 2011, and 2012) with rank fitted as a fixed effect.

#### Individual chick responses to treatment

Our first indicator of individual chick performance, growth rate during the linear phase of growth, correlates well in shags with prefledging mass (Reed et al. 2012), which studies in a range of bird species have shown to be positively related to recruitment probability (Magrath 1991; Schwagmeyer and Mock 2008). We obtained individual growth rates during this linear growth phase by fitting a linear regression for each chick. Each chick had 3–5 measurement points (mean 4.7, 1231 measurements on 259 chicks) apart from in 2007, when only two measurements per chick were possible (at treatment and toward the end of the linear growth phase, mean age 29.3 days). The data have previously been shown to be quantitatively robust to this restricted sampling (Reed et al. 2012).

We tested whether the rank-specific effect of treatment on growth rate varied with environmental conditions by fitting a three-way interaction term between rank as a three-level factor, treatment as a two-level factor, and productivity as a covariate. We compared the fit of the three-way interaction with models containing all of its subsidiary two-way interactions in turn and simultaneously. All models also included sex to account for the faster growth rate of males, which may make them more expensive to rear and hence more sensitive to their rank, levels of parasitism, and/or prevailing conditions (Daunt et al. 2001a; Reed et al. 2008a). Therefore, we compared the fit of all models fitted to three terms: sex as a main effect, a sex-by-rank interaction, or a sex-by-rank-by-productivity interaction. We also tested whether sex affected chicks' responses to treatment in the same way as we tested the role of rank. To examine whether rank-specific treatment responses were simply a result of mass differences between siblings at treatment, we fitted mass at treatment instead of rank and undertook the same model fitting and selection procedure.

We also investigated whether treatment affected chick survival between treatment and fledging (age ∼50 days, Snow 1960). We used the same models as for chick growth rate, with survival modeled as a binary response with binomial errors and a logit-link function.

#### Whole-brood growth rates

To test whether chick treatment affected the total amount of parental provisioning to the nest, we modeled whole-brood growth rate. We examined the effects of treatment and productivity as main effects and their interaction and included brood size at the end of the linear growth phase to account for nests in which chicks died after treatment (45 nests of 131 lost at least one chick during this period) and brood sex ratio (number of males divided by brood size), as whole-brood growth rates are likely to depend on the relative proportion of the two sexes because of sex-specific differences in growth rate.

## Results

### Worm burdens

In 2010, nematode egg counts before treatment did not differ between drug-treated and control shag chick groups (−0.2 ± 0.9, *χ* = 0.03, df = 1, *P* = 0.865), but 5–15 days after treatment, drug-treated chicks released significantly fewer eggs than control chicks (−3.7 ± 0.9; *χ* = 8.5, df = 1, *P* < 0.001). Chicks of different rank did not differ in their fecal egg counts before or after treatment (before, *χ* = 1.08, df = 4, *P* = 0.897; A chicks: 0.15 ± 0.11; B chicks: 0.09 ± 0.06; C chicks: 0.05 ± 0.05; after, rank-by-treatment interaction for both B and C chicks compared to A, *P* > 0.8; main effect of rank in addition to treatment, B and C chicks both *P* > 0.2 compared to A). Parasite burdens did not differ between years (as a categorical main effect, 2010–2012 only, *χ* = 1.58, df = 2, *P* = 0.453).

### Individual growth rates

**Figure 2:**
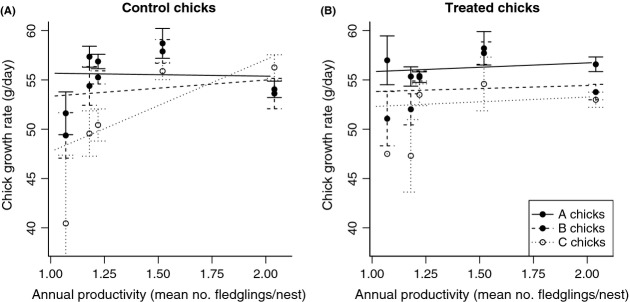
The growth rate of siblings of different ranks in naturally parasitized control broods (left panel) and in drug-treated broods (right panel) across a range of environmental conditions, represented by productivity. A chicks are shown with a black solid line and filled symbols, B chicks with a black long-dashed line and filled symbols, and C chicks with a fine-dashed line and open symbols. Points show mean values ±1 SE for each rank in each year, and the lines show the fitted relationship.

Different ranks responded differently to treatment, but this varied with productivity (Fig.2). Chick growth rate across the 5 years was best explained by a model including an interaction between rank, treatment, and productivity (interaction present in 3 of 4 models of equivalent best fit, Table2; three-way interaction, *P* = 0.005 in best-fit model). Neither productivity nor treatment affected the growth rate of older siblings. However, C chicks in naturally infected control broods grew faster in more productive years. C chick growth rate was also affected by antiparasite treatment, but the direction of this effect depended on productivity: treatment increased C chick growth rate in less productive years and decreased it in the most productive year. As a result, in treated broods, all chicks responded similarly to productivity (Fig.2). The effect of rank on the outcome of treatment was driven by the C chick (in best-fit model, interaction effect size compared to A chick: B chick −0.8 ± 2.7 g/day, *P* = 0.766; C chick −8.2 ± 2.9 g/day, *P* = 0.0005; Table2).

**Table 2:** The 10 best model fits explaining chick growth rate in descending order of fit, with a full description of the best-fit model. ΔAICs are relative to the best-fit model. In the model description, for brevity, parameter estimates and significances for rank terms are shown only for the C chick compared to the A; for B chick, main effect and all interactions *P* > 0.3.

Model terms	ΔAIC
Treatment * Rank * Prod. + Sex * Rank	0.0
Treatment * Rank * Prod. + Sex	0.7
Treatment * Rank * Prod. + Sex * Rank * Prod.	1.4
Rank * Prod. + Sex * Rank	1.7
Rank * Prod. + Sex	2.2
Treatment + Sex * Rank * Prod.	3.2
Treatment * Prod. + Sex * Rank * Prod.	3.4
Treatment * Rank + Treatment * Prod. + Rank * Prod. + Sex * Rank	5.5
Treatment * Rank + Treatment * Prod. + Rank * Prod. + Sex	6.0
Treatment * Rank + Sex * Rank * Prod.	7.0

Term	Estimate	Standard error	df	*t*	*P*
(Intercept)	53.9	2.7	214	20.07	0
Main effects					
Rank	−18.1	3.5	214	−5.19	0
Treatment	0.2	3.5	127	0.07	0.946
Productivity	0.4	1.8	127	0.24	0.810
Sex	2.0	0.8	214	2.38	0.018
Two-way interactions					
Rank * Treatment	12.1	4.5	214	2.67	0.008
Rank * Productivity	9.2	2.2	214	4.11	0.000
Treatment * Productivity	0.2	2.3	127	0.09	0.931
Rank * Sex	2.3	1.2	214	1.92	0.057
Three-way interaction					
Rank * Treatment * Productivity	−8.2	2.9	214	−2.82	0.005

The dependence of the treatment effect on rank was not simply a consequence of size differences at treatment. When the models in Table2 were fitted to mass at treatment instead of rank, the three-way interaction between dosing mass, productivity, and treatment was not present in any of the best-supported models (best-fitting model containing that interaction, ΔAIC = 3.9 from best fit) nor was the interaction significant (*F*_1,200_ = 1.51, *P* = 0.221).

Overall, males grew faster than females (55.8 ± 0.3 g/day compared to 53.1 ± 0.4 g/day; in best-fit model, *t* = 2.40, *P* = 0.017), and higher-ranked chicks grew faster than lower-ranked chicks (A chick: 55.8 ± 0.3 g/day, B chick: 54.5 ± 0.4 g/day, C chick: 52.6 ± 0.6 g/day; A compared to B, *t* = −0.86, *P* = 0.393; A compared to C, *t* = −5.20, *P* < 0.001). However, male and female chicks did not differ significantly in their response to productivity, treatment, or hatching order (all interactions with sex *P* > 0.05).

### Individual survival

Treatment did not affect survival from treatment to fledging in any year or for any rank (Table3). However, mortality was low overall, with only 59 of 458 chicks dying after treatment (32 controls, 27 drug-treated). C chicks were less likely to survive to fledging irrespective of treatment (main effect of rank, *P* < 0.001), and all brood members had greater survival from treatment to fledging in more productive years (main effect of productivity, *P* = 0.002). Sex did not significantly affect post-treatment survival to fledging (main effect and all interactions, *P* > 0.07).

**Table 3:** The 10 best model fits explaining chick survival in descending order of fit, with a full description of the best-fit model. ΔAICs are shown relative to the best-fit model. In the model description, for brevity, parameter estimates and significances for rank terms are shown only for the C chick compared to the A; for B chick, main effect and all interactions *P* > 0.2.

Model	ΔAIC
Rank * Treatment + Prod. + Sex	0.0
Treatment * Prod. + Rank + Sex	0.8
Rank + Sex + Prod. + Treatment	1.6
Rank * Treatment + Prod. + Sex * Rank	4.0
Rank * Treatment + Rank * Prod. + Treatment * Prod. + Sex	4.2
Treatment * Prod. + Rank + Sex * Rank	4.9
Rank * Prod. + Treatment + Sex	5.4
Rank + Sex * Rank + Prod. + Treatment	5.5
Rank * Treatment + Rank * Prod. + Treatment * Prod. + Sex * Rank	8.3
Rank * Treatment * Prod. + Sex	8.3

Best fit model: Term	Estimate	Standard error	*Z*	*P*
(Intercept)	0.1	1.4	0.10	0.923
Main effects				
Rank	−3.4	1.0	−3.47	0.001
Treatment	0.8	1.8	0.45	0.654
Productivity	3.0	0.9	3.55	0.000
Sex	1.2	0.5	2.31	0.021
Interactions				
Rank * Treatment	−1.6	1.8	−0.88	0.380

### Whole-brood growth rates

**Figure 3:**
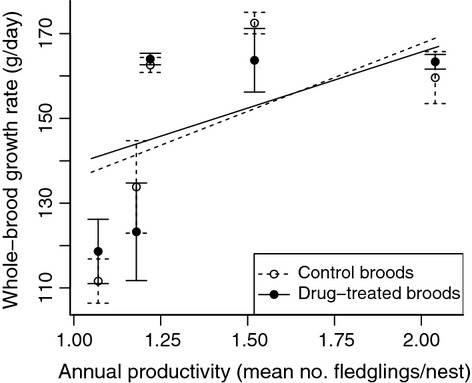
Whole-brood growth rate in relation to productivity for drug-treated and control broods. Control broods are shown with a dotted line and open symbols and antiparasite-treated broods with a solid line and filled symbols. Points show mean values ±1 SE, and lines show the fitted relationship.

The combined growth rate of the whole brood was not affected by treatment, and there was no significant interaction between treatment and productivity (Fig.3, Table4). Overall, broods grew more slowly in less productive years, and larger broods grew faster (Table4).

**Table 4:** All models tested to explain whole-brood growth rate, in order of decreasing fit, with a full description of the best-fit model. ΔAICs are in relation to the best-fit model.

Model	ΔAIC
Productivity + Brood size	0.0
Productivity + Brood size + Sex ratio	0.9
Treatment + Productivity + Brood size	1.9
Treatment * Productivity + Brood size	3.8
Treatment * Productivity * Sex ratio + Brood size	7.3
Brood size + Sex ratio	10.4
Brood size * Sex ratio	11.0
Treatment * Sex ratio + Brood size	13.6

Best fit model Term	Estimate	Standard error	*t*	*P*
Brood size	25.8	3.4	7.58	<0.001
Productivity	16.7	5.0	3.34	0.001

## Discussion

In this study, we have shown that quantitative measures of prevailing environmental conditions can explain variation in responses to parasitism. Moreover, individuals can differ substantially in how parasitism and environmental conditions interact to shape their juvenile development. We found that last-hatched chicks generally responded more strongly than their older siblings to both antinematode treatment and our proxy for environmental conditions. However, the relationship between hatching order, parasitism, and environmental conditions was not simply additive: In the most productive year, parasite treatment had the opposite effect to other years and decreased the growth rate of youngest siblings. These effects were likely due to inherent differences between brood mates in physiology or competitive ability, as siblings did not differ in their parasite load. We found no evidence that parents altered overall investment in drug-treated compared to control broods, as treatment did not change the growth rate of the whole brood. However, as treatment altered the relative growth rates of individuals within the brood, it may have affected how parents adjust allocation of resources among brood members. Overall, our results show that parasitism is important in driving between-individual variation in juvenile developmental trajectories, which could have lifelong fitness consequences (Lindström 1999; Metcalfe and Monaghan 2001; Monaghan 2008).

The quantitative relationship we found between environmental conditions and parasite effects demonstrates the importance of repeating experimental manipulations across a range of natural conditions. Beyond simply demonstrating the generality of findings, such repeats enable us to account informatively for differences in treatment effects. Although many studies observe that wild hosts' responses to infection can vary between years and seasons, or when environmental variables such as food availability are experimentally manipulated (e.g., Howe 1992; Stien et al. 2002; Redpath et al. 2006; Brzek and Konarzewski 2007; O'Brien and Dawson 2008; Pedersen and Greives 2008; Knowles et al. 2010b), we know of no previous study that quantitates the effect of prevailing environmental conditions and incorporates such measures to better explain how individual hosts are impacted by parasites in the wild. Our quantification was informative even with only a limited number of experimental years, a common constraint in wild systems. Importantly, our results show that the interplay between parasitism and environmental conditions was not an intuitively simple case of steadily decreasing parasite impacts as conditions improved. Rather, treatment had the opposite effect in the most productive year to the less productive years, with last-hatched chicks in control broods in the most productive year growing faster than their older siblings, indicating that dealing with an infection may trade off against growth in complex ways. This observation suggests a little explored aspect of the role of younger siblings as highly plastic “resource-tracking” offspring. They may be adaptive for parents not only by minimizing costs of misplaced investment in poor conditions (Mock and Forbes 1995; Forbes et al. 2002; Forbes 2009) but also by taking advantage of exceptionally favorable conditions through a more plastic growth rate, maximizing parental fitness in high-productivity seasons. This high-risk, high-return strategy is considered in terms of mortality by Forbes (2009, 2011) and applied to an observational dataset, but we are not aware of any theoretical or empirical extension such as ours of the high-return aspect of this theory.

Our results highlight the role that parasitism may play in brood reduction and family conflict. Based on fecal egg counts, we found no evidence for systematic variation between siblings in their initial worm burdens. This suggests that heterogeneity among siblings in their response to treatment stems from inherent differences between brood members rather than differences in infection levels. Siblings may experience differing costs of a given infection for two reasons, which could operate simultaneously. First, brood members may differ intrinsically in how they cope with both parasite infection and the prevailing external conditions. This may arise from inherent differences such as their relative size and physiology at hatching (Mock and Forbes 1995; Mock and Parker 1997; Bonabeau et al. 1998; Drummond 2006), within-brood differences in maternal allocation of antibodies to eggs (Pihlaja et al. 2006; Hasselquist and Nilsson 2009; Martyka et al. 2011), or differences in parental provisioning early in life (Parker et al. 2002). Second, parasitism may alter competitive dynamics within the brood. If the impact of infection and prevailing environmental conditions affect chicks' competitive abilities in different ways, within-brood interactions may have a different outcome. These influences on individual chicks' competitive environment may lead to siblings effectively inhabiting different worlds despite growing up in the same nest (Forbes 2011). Indeed, mechanisms that have evolved to give C chicks a developmental boost in their harsher social environment may also give them more potential to do well in benign conditions, as we found.

Although within-brood development patterns were influenced by antiparasite treatment, we found no evidence that treatment influenced total parental investment in the brood, which contrasts with similar studies in ectoparasite systems, where increases in provisioning to parasitized broods have been reported (Christe et al. 1996; Hurtrez-Boussès et al. 1998). However, in ectoparasite systems, changes to provisioning may be a response to a change in parents' own parasite load, altered by the ectoparasites redistributing themselves among the family after manipulation of chick parasite load (Bize et al. 2004; Fitze et al. 2004; Gallizzi et al. 2008). It is therefore difficult in ectoparasite systems to isolate specific parental responses to chick infection levels. Endoparasite systems, on the other hand, allow us to exclude this possibility by using a trophically transmitted parasite that, to our knowledge, cannot be passed from chicks to parents.

In summary, we have demonstrated that parasite infection is an important component of juvenile shags' developmental environment whose impact on different brood members depends on the prevailing environmental conditions. Infection during early life may have substantial consequences for an individual's future success as juveniles are more susceptible than adults to infection and its effects (Hudson and Dobson 1997; Møller 1997; Wakelin & Apanius 1997; Sol, Jovani & Torres 2003) and conditions during development can have lifelong fitness consequences (Lindström 1999; Metcalfe and Monaghan 2001; Monaghan 2008). Our results not only demonstrate the importance of considering both environmental and individual variability when assessing the role of parasites in host ecology, but also show that a quantitative consideration of prevailing conditions can be valuable in understanding individual responses to experimental manipulations in wild systems.

## Data accessibility

All data used in the analyses in this paper have been placed in the Dryad repository, DOI 10.5061/dryad.7sn14, with the exception of the productivity data, which are online at: Newell, M., Harris, M., Wanless, S., Burthe, S., Bogdanova, M., Gunn, C., and Daunt, F. (2012). The Isle of May Long-Term Study (IMLOTS) Seabird Annual Breeding Success 1982–2012. NERC Environmental Information Data Centre. doi: 10.5285/d38b609b-7bc1-4204-86dd-022375208d4f.
